# Preliminary Assessment of Chinese Strategy in Controlling Reemergent Local Outbreak of COVID-19

**DOI:** 10.3389/fpubh.2021.650672

**Published:** 2021-07-02

**Authors:** Zhouhan Wang, Yanqi Jin, Xi Jin, Yingfeng Lu, Xiaopeng Yu, Lanjuan Li, Yimin Zhang

**Affiliations:** ^1^State Key Laboratory for Diagnosis and Treatment of Infectious Diseases, Department of Infectious Diseases, National Clinical Research Center for Infectious Diseases, Collaborative Innovation Center for Diagnosis and Treatment of Infectious Diseases, The First Affiliated Hospital, College of Medicine, Zhejiang University, Hangzhou, China; ^2^Department of Gastroenterology, The First Affiliated Hospital, College of Medicine, Zhejiang University, Hangzhou, China; ^3^Department of Infectious Diseases, The First Affiliated Hospital, College of Medicine, Zhejiang University, Hangzhou, China

**Keywords:** COVID-19, SARS-CoV-2, reemergent local outbreak, asymptomatic infection, China, GDP

## Abstract

Reemergent local outbreaks of coronavirus disease 2019 (COVID-19) have occurred in China, yet few Chinese response strategies and its evaluation have been reported. This study presents a preliminary assessment of Chinese strategy in controlling reemergent local outbreaks of COVID-19. Time course of accumulative and daily new cases and time-varying reproductive numbers (Rt) of outbreak areas were presented. The asymptomatic rate, days required to control the outbreaks, seeding time (ST), and doubling time (DT) of areas with over 96 reemergent cases were calculated. National and local year-on-year growth rates of gross domestic product (GDP) were presented. Accumulative numbers of 30, 8, 11, 430, 15, 139, 1,067, 382, 42, and 94 confirmed reemergent COVID-19 cases were diagnosed in Hulun Buir, Shanghai, Tianjin, Kashgar, Qingdao, Dalian, Urumchi, Beijing, Jilin, and Harbin, respectively. Among them, maximum rate of asymptomatic infections was 81.9%. Time required to control the local outbreaks in the areas given above varied from 29 to 51 days. After activation of outbreak responses, the late-stage DTs of Kashgar, Urumchi, Beijing, and Dalian were apparently lengthened compared to the early-stage DTs. Although the year-on-year GDP growth rate of Urumchi was slightly affected, the GDP growth rate of Dalian, Beijing, Jilin, and Harbin kept rising during the reemergence. Moreover, the year-on-year GDP growth rate of Mainland China turned positive regardless of the reemergent local outbreaks. In general, the Chinese strategy to maintain the status of no or minimal transmission was effective in balancing the control of COVID-19 reemergent local outbreak and the recovery of economy.

## Introduction

According to the WHO, 80,453,105 people were diagnosed with coronavirus disease 2019 (COVID-19) and 1,775,776 of them died as of December 30, 2020 (1). According to Michael Ryan, the Executive Director of WHO Health Emergencies, China, however, has reached “extremely low levels of the virus” (2). Several reemergent local outbreaks were reported in Hulun Buir, Shanghai, Tianjin, Kashgar, Qingdao, Dalian, Urumchi, Beijing, Jilin, and Harbin and had been later controlled. However, few Chinese strategies for tackling reemergent local outbreaks were reported. In this study, the time course of these local reemergent outbreaks was revealed, and a preliminary assessment of Chinese strategy in facing local reemergent outbreaks was presented.

## Methods

Updates of COVID-19 cases in Hulun Buir, Shanghai, Tianjin, Kashgar, Qingdao, Dalian, Urumchi, Beijing, Jilin, and Harbin were extracted from the situation reports of the official websites of local governments (3–11). Imported cases were excluded in this study. According to the “WHO COVID-19: Case Definitions” (12), a person with laboratory confirmation of COVID-19 infection, irrespective of clinical signs and symptoms, is defined as a confirmed case. Based on the Protocol for Prevention and Control of COVID-19 (Edition 6) (13), cases that resulted positive for reverse transcriptase (RT)-PCR or antibody test but without clinical syndrome were defined as cases with asymptomatic infections. People who presented with asymptomatic infection at first but later developed clinical syndrome were excluded from the asymptomatic cases. All cases were reported within 2 hours of diagnosis. Accumulated numbers of confirmed cases from the 1st day a case is reported to the 14th day with no new case of outbreaks reported in the areas mentioned above, which was regarded as the time required to control an outbreak, were presented as epidemiologic curve. Cases with identical origin as the originating site were counted together in the originating site cases. A number of daily new symptomatic and asymptomatic cases of reemergences as mentioned above were presented as a bar graph. Curves of time-varying reproduction number (Rt) were generated using the EpiEstim R package (14). The asymptomatic rate, the number of people received RT-PCR test in the first round of mass testing, and the number of people received first-round RT-PCR test per day are presented in a table.

According to the study of Zhou L (15), 30 countries meeting the following criteria were selected to structure the seeding time (ST)/doubling time (DT) model: (a) having over 5,000 cases as of March 31, 2020 or (b) having over 40 case-reporting days and 100 cases between the first case-reported day and March 31, 2020. Then, two specialists of the Chinese Center for Disease Control and Prevention independently determined the “takeoff” date of each country. When a disagreement occurred, a third specialist was involved, and discussion was conducted until the research team met a consensus. The accumulative number of cases on the day before the “takeoff” date was obtained and defined as seeding number (SN). The time interval between the date of the first reported case and the date of the a number of cases reaching the SN of an area was defined as ST. DT was the time interval required to double the accumulative number of cases in an area. For example, DT1 refers to the time interval between the confirmed cases that reached SN and 2 × SN, and DT2 refers to the time interval between the confirmed cases that reached 2 × SN and 4 × SN, etc. DT1 ~ DT3 were defined as early-stage DTs, and DT4 ~ DT6 were defined as late-stage DTs (15). Then, the median SN of the 30 countries mentioned above was calculated (median SN = 12) and was used to generate ST/DT model. To at least fully present the early-stage DTs, the data of four cities (i.e., Kashgar, Dalian, Urumchi, and Beijing) that reached the threshold of 96 cases were shown as a scatter diagram. Year-on-year growth rates of gross domestic product (GDP) of Dalian, Urumchi, Beijing, Jilin, Harbin, and Mainland China were extracted from the official websites of the government statistical bureau and presented as a bar graph (16–21). The statistical scopes of GDP are prefecture cities or municipalities directly under the central government where the local reemergent outbreak occurred. Data of other areas were not shown since the local GDP at the time of reemergences had not been counted.

Informed consent from an individual was not required in the presence of a public health emergency. The Clinical Research Ethics Committee of the First Affiliated Hospital, College of Medicine, Zhejiang University, Hangzhou, China, approved this study.

## Results

Accumulative numbers of 30, 8, 11, 430, 15, 139, 1,067, 382, 42, and 94 confirmed cases were diagnosed in Hulun Buir, Shanghai, Tianjin, Kashgar, Qingdao, Dalian, Urumchi, Beijing, Jilin, and Harbin, respectively (Table 1). Asymptomatic infections of local reemergent areas mentioned above, respectively, account for 6.7% (2/30), 0% (0/8), 18.2% (2/11), 81.9% (352/430), 6.7% (1/15), 29.5% (41/139), 22.5% (240/1067), 12.3% (47/382), 4.8% (2/42), and 18.1% (17/94) of all confirmed cases (Table 1). The first-round mass RT-PCR test of SARS-CoV-2 in Hulun Buir, Tianjin, Kashgar, Qingdao, Dalian, Urumchi, and Beijing had been carried out within 3, 4, 3, 5, 9, 10, and 21 days from the onset of the first case (Table 1). Data of Harbin were unavailable and thus not shown in the table.

**Table 1:** Cases and testing data of local reemergent areas.

	**Time interval**	**Confirmed cases^**a**^, persons**	**Symptomatic cases, persons**	**Asymptomatic cases^**b**^, persons (%^**c**^)**	**Number of people receiving 1st round PCR test**	**Number of people receiving 1st round PCR test per day^**d**^**
Hulun Buir	November 21–December 23, 2020	30	28	2 (6.7)	203,326	67,775
Shanghai^e^	November 9–December 7, 2020	8	8	0 (0)	41,852	3,219
Tianjin	November 8–December 7, 2020	11	9	2 (18.2)	2,467,411	616,852
Kashgar	October 24–November 21, 2020	430	78	352 (81.9)	4,746,500	1,582,166
Qingdao	September 24–November 12, 2020	15	14	1 (6.7)	10,430,000	2,086,000
Dalian	July 22–August 19, 2020	139	98	41 (29.5)	3,892,000	432,444
Urumchi	July 15–August 31, 2020	1,067	827	240 (22.5)	2,309,537	230,953
Beijing	June 11–July 31, 2020	382	335	47 (12.3)	10,414,000	495,904
Jilin^*e*^	May 7–June 6, 2020	42	40	2 (4.8)	88,303	7,358
Harbin	April 9–May 23, 2020	94	77	17 (18.1)	N/A	N/A

a *Cases with identical origin as the originating site were counted together in the originating site cases.*

b *People who presented with asymptomatic infection at first but later developed clinical syndrome were excluded from the asymptomatic cases.*

c Asymptomatic rate = (Asymptomatic cases/Confirmed cases) ^*^100%.

d *Number of people receiving first-round PCR test per day = Number of people receiving first-round PCR test/Days required for first-round mass testing*.

e *Shanghai and Jilin have only tested the close contacts and close contacts of close contacts due to the early detection*.

**Figure 1:**
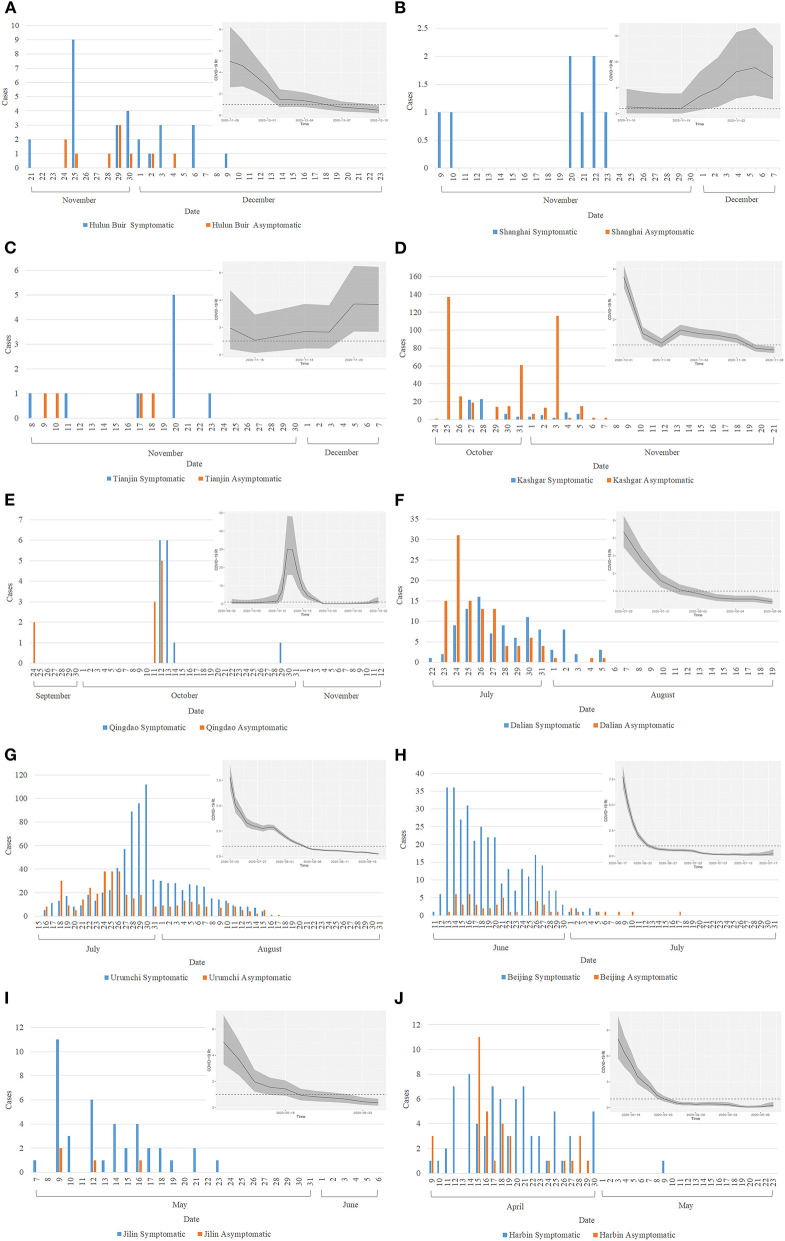
Daily new symptomatic and asymptomatic cases and time-varying reproductive numbers (Rt) curve in reemergent areas. Bar graphs present daily new cases of the local reemergent outbreaks in Hulun Buir **(A)**, Shanghai **(B)**, Tianjin **(C)**, Kashgar **(D)**, Qingdao **(E)**, Dalian **(F)**, Urumchi **(G)**, Beijing **(H)**, Jilin **(I)**, and Harbin **(J)**. Rt curves of reemergent areas are shown on the upper right part of each bar graph, respectively. Cases with identical origin as the originating site were counted together in the originating site cases. Rt stands for time-varying reproductive number.

**Figure 2:**
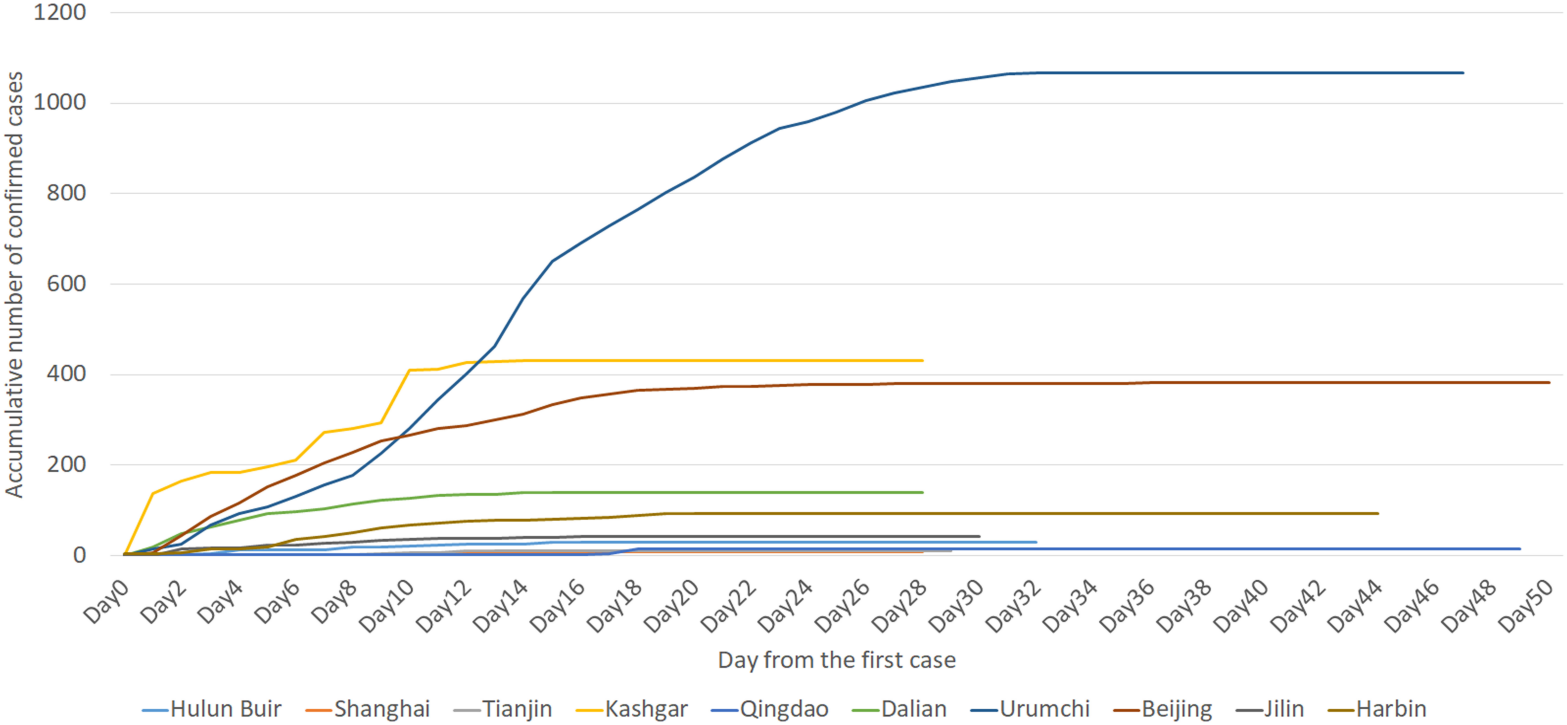
Time course of the accumulated number of confirmed cases in reemergent areas. This epidemiological curve shows an accumulative number of confirmed cases of Hulun Buir, Shanghai, Tianjin, Kashgar, Qingdao, Dalian, Urumchi, Beijing, Jilin, and Harbin. The curve was presented by day, from the day the 1st case was reported to the 14th day with no new case reported. People who presented with asymptomatic infection at first but later developed clinical syndrome were excluded from the asymptomatic cases.

**Figure 3:**
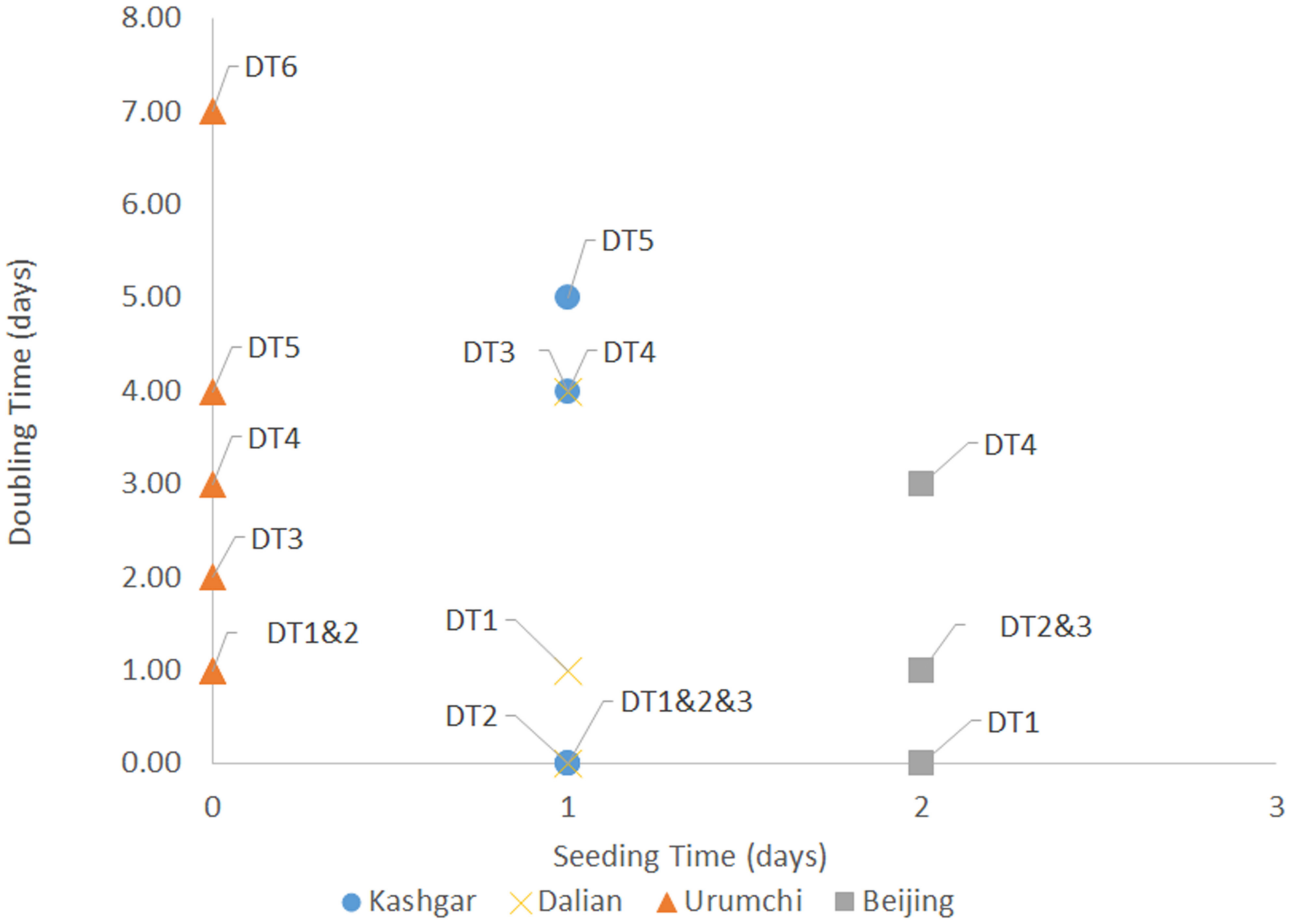
Seeding time (ST) and doubling time (DT) of Kashgar, Dalian, Urumchi, and Beijing reemergent outbreaks. This scatter diagram shows ST and DT1~6 of Kashgar, Dalian, Urumchi, and Beijing reemergent outbreaks. The time interval between the date of the first reported case and the date of a number of cases reaching the median seeding number (SN) of an area (median SN = 12 according to the study of Zhou L) was defined as ST. DT was the time interval required to double the accumulative number of cases of an area. DT1 ~ DT6 were calculated according to the time interval between the 12 and 24, 24 and 48, 48 and 96, 96 and 192, 192 and 384, and 384 and 768 confirmed cases, respectively. ST stands for seeding time. DT stands for doubling time.

**Figure 4:**
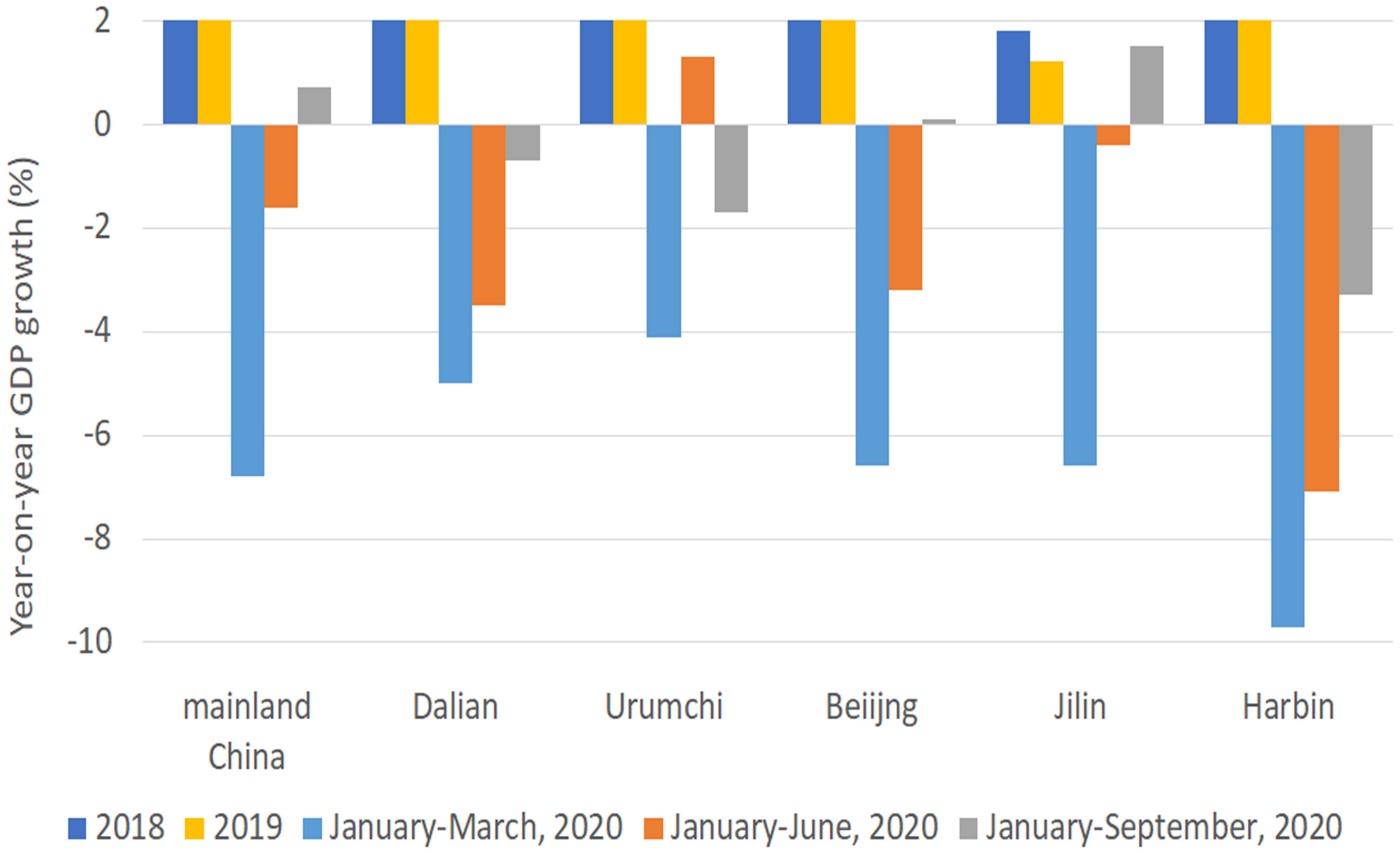
National and local year-on-year GDP growth rate. This bar graph presents the year-on-year GDP growth rate of Mainland China and the local year-on-year GDP growth rates of Dalian, Urumchi, Beijing, Jilin, and Harbin of 2018, 2019, and the first three quarters of 2020. GDP stands for gross domestic product.

The time required to control outbreaks in Hulun Buir, Shanghai, Tianjin, Kashgar, Qingdao, Dalian, Urumchi, Beijing, Jilin, and Harbin were 33, 29, 30, 29, 50, 29, 48, 51, 31, and 45 days, respectively (Figures 1, 2). According to the previously established model of Zhou L, ST and early-stage DTs of Kashgar, Dalian, Urumchi, and Beijing revealed high escalating probability (15). After the activation of outbreak responses, the late-stage DTs were apparently lengthened compared to the early-stage DTs (Figure 3). Local year-on-year GDP growth rates of Dalian, Beijing, Jilin, and Harbin were continuously rising regardless of the local reemergent outbreaks. Despite the GDP growth rate of Urumchi was slightly affected during the reemergent period, a positive trend can still be recognized compared to the complete lockdown period (January 2020–March 2020). GDP of Mainland China turned positive regardless of the reemergent local outbreak (Figure 4).

## Discussion

With the rising number of cases according to the situation reports of WHO, the COVID-19 pandemic seems possible to continue for a longer period. COVID-19 pandemic has seriously affected the global economy, and according to Ms. Gopinath on the October 2020 World Economic Outlook Press Briefing, China may be the only major economy to achieve positive output growth this year (22). Although the overall level of SARS-CoV-2 transmission in China was close to zero, reemergent local outbreaks were still occurring. In this situation, how China strikes a balance between controlling outbreaks and revitalizing the economy was critical to pulling the world out of the deep economic recession. Emphases of Chinese strategy in tackling reemergent local outbreak included a rapid response to local reemergent, reasonable test range covering asymptomatic infections, precise region management according to different transmission risk, and inspection of inbound persons and imported cargoes (13). At the present stage, the goal of Chinese strategy is to maintain the status of no or minimal transmission until a safe and effective vaccine is available for everyone (23). Such strategy was able to well-manage the balance between outbreak control and economic recovery.

Firstly, a retrospective epidemiological investigation was carried out as soon as the first case was reported in a certain region. Close contacts and close contacts of close contact were found, and RT-PCR testing was immediately conducted. If the outbreak has an unclear source or a wide affected region, mass testing covering all citizens in related regions was rapidly conducted. According to the previous studies, asymptomatic infected persons can transmit SARS-CoV-2 to others and they account for approximately 40~50% of all cases (24). In this study, the asymptomatic rate of the local reemergent outbreaks that occurred in China between April 2020 and December 2020 was up to 81.9%, indicating that the testing only for the symptomatic population was not enough. Chinese strategy of applying epidemiological investigation and a reasonable range of tests, sufficient to cover asymptomatic infections, was key to successfully control the local outbreaks.

Second, region management required flexible risk levels to be determined according to the population and epidemic status of certain districts, and a nationwide joint control strategy (JCS) was applied based on the assessment of risk level. According to the JCS (13), related regions were immediately upgraded to moderate- or high-risk areas as soon as the first case was reported. Corresponding management was applied to people in those areas and people traveled from those areas within 14 days. Such strategy ensured that no secondary outbreak was triggered outside the initial reemergent area. With gaining experience, the delineation of areas under risk was more and more fine. Local reemergent cases had also appeared in Shenyang, Dalian, Beijing, Chengdu, and Mudanjiang in December 2020. A similar strategy has been applied, and the situation has been effectively contained up to the submission date.

Finally, an inspection of imported subjects, including inbound persons and cargoes, has always been one of the important measures for outbreak control in China. Chinese researchers extrapolated that the source of the local outbreak in Harbin in April was a student returning from overseas (25). Reemergence in Jilin in May was suspected to be associated with a possible importation event, and the result of viral genome sequencing strongly suggested that the first case is related to the COVID-19 virus imported from Europe (26). Sources of early-stage local reemergence were mostly associated with people who traveled from outside China. However, subsequent local reemergences in Beijing, Urumchi, Dalian, Qingdao, and Tianjin may be related to imported cold-chain packages to various degrees. Among them, environmental swab samples of imported cold-chain packages in Beijing and Dalian reemergences were tested nucleic acid positive for SARS-CoV-2 (27, 28). Furthermore, the SARS-CoV-2 virus was directly isolated from the surface of imported cold-chain cargoes in Qingdao (29). Similarly, the source of the local reemergence in Tianjin may be frozen pig heads from North America (30). These results were highly suggestive that SARS-CoV-2 may survive for a long time on cold-chain cargoes and trigger transmission. Nevertheless, research studies have shown that environmental temperature is related to the stability of SARS-CoV-2 (31). In the local reemergences in Kashgar in October and Shanghai in November, the transmission source may be both cold-chain and non-cold-chain containers imported from overseas (32, 33). These two local reemergences suggested that with the arrival of winter, SARS-CoV-2 may survive longer on non-cold-chain-transported goods and may also cause virus transmission. Considering the severe situation in the countries outside of China, the management of incoming items requires special attention. China has set up a technical guide aiming at prevention, control, and disinfection of cold chain (34). Non-cold-chain cargoes have also gained the attention of the government, and Zhejiang Province was the first to publish an emergency plan (35). Chinese prevention and control strategy has gradually become more comprehensive from the management of inbound people from outside China to the management of imported cold-chain and even non-cold-chain cargoes.

Regarding the effect of such comprehensive strategy, late-stage DTs of Kashgar, Urumchi, Beijing, and Dalian were lengthened compared to early-stage DTs, indicating the effectiveness of local emergency control. Time course also revealed that such a strategy was able to control reemergent outbreaks within 51 days. The success of such a strategy has also been proven by the national and local GDP. According to the year-on-year GDP growth rate data of the past 2 years, the national and local GDP of China has been in a state of relatively stable growth. Therefore, the COVID-19 pandemic and reemergent local outbreak may be regarded as the biggest variable during the first three quarters of 2020 that may affect GDP. As of the first three quarters of 2020, the year-on-year growth rate of local GDP in reemergent areas turned positive in spite of the local reemergences under such a containing strategy. Despite the GDP of Urumchi was slightly affected during the reemergence period, a positive trend can still be recognized. GDP of Mainland China turned positive regardless of the reemergent local outbreak (Figure 4). Comprehensive means were able to efficiently suppress the transmission, and therefore, basic service and production can return to normal. Moreover, it is worth mentioning that GDP may be affected by small-scale sporadic cases that appeared in a district or block when a certain area enters the relatively stable stage. Although there may be few confirmed cases, the cost of preventing contagion may still be enormous. Taking Ruili as an example, only two cases were confirmed in September 2020, but the city paid the price of a 7-day lockdown. Such measures are necessary but may exert greater influence on the local economy.

This research study has several limitations. Firstly, in our study, further analysis involving demographic features, social relationships, and traveling history of the confirmed cases remained difficult to implement due to the limited open-access data, which hindered further epidemiologic analysis such as the reconstruction of transmission pairs (36). Secondly, the GDP of a certain place is regulated by various factors such as the local economic structure, economic development level, and economic policies. It is almost impossible to completely remove the confounders that affect GDP. Finally, the year-on-year GDP growth rate alone may not be a comprehensive assessment of the influence on the economy. Other evaluation indexes with respect to social and/or economic influence were not discussed due to the limitation of data resources. Further research studies are needed to better understand the factors that influence the containment of the pandemic and local reemergent outbreaks and their relationship with the economy and society.

## Conclusions

Chinese strategy of rapid response to local reemergent, reasonable test range covering asymptomatic infections, precise region management according to different transmission risk, and inspection of inbound persons and imported cargoes was effective in balancing the control of reemergent local outbreak and the recovery of economy. The possibility of object–human transmission requires more attention in controlling local outbreaks, especially in winter.

## Data Availability Statement

The original contributions presented in the study are included in the article/supplementary material, further inquiries can be directed to the corresponding author/s.

## Ethics Statement

The studies involving human participants were reviewed and approved by the Clinical Research Ethics Committee of the First Affiliated Hospital, College of Medicine, Zhejiang University. Written informed consent from the participants' legal guardian/next of kin was not required to participate in this study in accordance with the national legislation and the institutional requirements.

## Author Contributions

ZW, YJ, and XJ wrote the manuscript. XY, YL, YZ, and LL read and edited the manuscript. All authors contributed to the article and approved the submitted version.

## Conflict of Interest

The authors declare that the research was conducted in the absence of any commercial or financial relationships that could be construed as a potential conflict of interest.
